# Enhancement of methane production from Cotton Stalk using different pretreatment techniques

**DOI:** 10.1038/s41598-018-21413-x

**Published:** 2018-02-22

**Authors:** Han Zhang, Zhifang Ning, Habiba Khalid, Ruihong Zhang, Guangqing Liu, Chang Chen

**Affiliations:** 1 Biomass Energy and Environmental Research Center, College of Chemical Engineering, Beijing University of Chemical Technology, Beijing, 100029 China; 2 College of Life Science and Technology, Beijing University of Chemical Technology, Beijing, 100029 China; 3 Department of Biological and Agricultural Engineering, University of California, Davis, CA 95616 United States

## Abstract

China produces large amount of cotton stalk (CS) residues as agricultural biomass, which are incinerated on-site, causing air pollution. The high organic content of CS could be utilized for biogas production, but the direct digestion without pretreatment always leads to a low methane yield and biodegradability, due to the complicated structure of lignocellulose. In order to search best fitting pretreatment methods in effective anaerobic digestion (AD) of CS, effects of various pretreatments including KOH, NaOH, Ca(OH)_2_, alkali hydrogen peroxide (AHP), H_2_SO_4_, H_3_PO_4_ and steam explosion (SE) were studied. It was seen that all treatments resulted in varying methane yields. Among all the pretreatments, acid pretreatment is not suitable for AD of CS. The results showed that the highest cumulative methane yield (CMY) of 192.4 mL·gVS^−1^ was obtained after 3% AHP pretreatment of CS, and the methane yield improved by 254.3% than the untreated CS. Therefore, AHP treatment was proven to be an efficient pretreatment technique. XRD and FTIR analyses had shown that pretreated CS had favorable structural changes. This research is beneficial in developing environment friendly and cost-effective pretreatment technologies to utilize CS for methane production in future application.

## Introduction

Cotton is an important economic crop in China which has output accounting for 25% of the total world’s cotton production^1^. The cultivation of cotton results in the wastes of cotton stalk (CS) amounting 40 million tons annually^2^. The large amount of CS is used as fuel for cooking and has adverse environmental impacts such as air pollutants emission in rural China. It is necessary to use a cleaner and greener method to treat CS.

CS contains amount of carbohydrates, like cellulose and hemicelluloses, which can be converted into a variety of usable forms of energy such as bioethanol, biohydrogen, and biogas^3,4^. Among all the biofuels, biogas is paid increasing attention because its production process and conditions are simpler than the production of other biofuels, which make biogas production more practical^5^. In general, anaerobic digestion (AD) is a three stage process: hydrolysis, acidogenesis and methanogenesis^6^. The hydrolysis reaction is considered to be the rate limiting step in the whole AD process due to the complicated structure of lignocellulosic biomass^6^. High cellulose and hemicellulose contents of CS makes it a potential feedstock for biogas production, but it forms a recalcitrant lignocellulose complex with lignin to limit the effective conversion to methane. Especially, the presence of lignin in lignocellulosic CS residues serves as a protective shield and restricts the enzymatic hydrolysis of cellulose and hemicellulose to dissolved organic matter^7^. Therefore, an effective pretreatment step is required to accelerate the hydrolysis process and enhance the digestibility of CS.

Chemical pretreatment (acid, alkali, organic solvent, oxidation, catalyzed steam explosion etc.)^8–11^, physical pretreatment (comminution, liquid hot water, steam explosion, irradiation, etc.)^12^, and biological pretreatment^1^ techniques have been investigated in previous research of lignocellulosic biomass. Among all the pretreatment methods, chemical treatment is the most common and intensively studied method for lignocellulosic biomass in AD process. The widely used reagents include sodium hydroxide (NaOH)^3,13,14^, calcium hydroxide (Ca(OH)_2_)^3,15,16^, potassium hydroxide (KOH)^16^, sulfuric acid (H_2_SO_4_)^3,9^, hydrochloric acid (HCl)^3^, phosphoric acid (H_3_PO_4_)^9,14,17^. For example, Sambusiti *et al*. reported that wheat straw treated by 10% NaOH (g/g TS) at 40 °C for 24 h achieved a 47% increase in the methane production^8^. Moreover, alkali hydrogen peroxide (AHP) pretreatment has gained popularity in recent years, because it is one of the most promising chemical method for the removal of lignin^18^. Sun *et al*.^18^ compared five stalks (wheat, rice, maize, rape and cotton stalks) by 2% AHP pretreatment for improving methane yield, and the methane yield of CS improved 55.7% than the untreated CS. As for physical pretreatment, steam explosion (SE) is also an attractive option. Li *et al*.^12^ found that the cumulative methane yield (CMY) of SE pretreated corn stalk at 1.2 MPa for 10 min could improve 63.9% of methane yield compared with untreated corn stalk. Although many researchers have investigated crop straw, the effects of various pretreatments differ considerably, therefore, the most economical and effective pretreatment method has not been fully explored. Additionally, few reports have shown the pretreatment effects on CS for improving methane yield. Such information is important for evaluation of pretreatments and their practical application. Therefore, a systematic study regarding the impacts of different pretreatment methods on CS for methane production is highly demanded.

The objectives of this research were to: (1) carry out KOH, NaOH, Ca(OH)_2_, AHP, H_2_SO_4_, H_3_PO_4_, SE pretreatment to evaluate their varying effects on CS, (2) systematically and comprehensively compare the AD performance of CS after different pretreatments, (3) verify structural changes of different pretreated CS.

## Methods

### Substrates

CS was obtained from a farm in Henan province, China. The raw CS was cut with a chaff-cutter to less than 5 cm in length and milled to 18-mesh powder by an high-speed grinder (XINGSHILIHE, Beijing, China), and then was put in airtight plastic bag at room temperature for future use. The inoculum sludge was collected from an anaerobic digester at Donghuashan biogas plant in Beijing, China.

### Pretreatment

The pretreatment contained the solutions of KOH (1.5, 3.0, 4.5, and 6.0%, w/w), NaOH (1.5, 3.0, 4.5, and 6.0%, w/w), Ca(OH)_2_ (1.0, 2.0, 3.0, and 4.0%, w/w), AHP (1.0, 2.0, 3.0, and 4.0%, w/w), H_2_SO_4_ (1.0, 2.0, 3.0, and 4.0%, w/w), H_3_PO_4_ (1.0, 2.0, 3.0, and 4.0%, w/w). 50 g of raw CS were added to the respective solutions. The moisture content (MC) was 90%, calculated using Eq. (1)^19^. All samples were pretreated at room temperature for 24 h and manually mixed once every four hours. The pretreated samples were rinsed with distilled water until neutral pH was attained, and the extra water was removed. SE pretreatment was performed in a high-pressure reactor. 80 g of CS were treated with saturated steam at 0.9, 1.2, 1.5 MPa and 5, 10, 15 min respectively. All pretreated CS were stored at 4 °C before experimentation.

1 $$\text{MC}( \% )=(1-\frac{{\rm{dry}}\,{\rm{matter}}\,{\rm{weight}}\,{\rm{of}}\,{\rm{CS}}}{{\rm{weight}}\,{\rm{of}}\,{\rm{CS}}+{\rm{water}}\,{\rm{added}}})\times 100 \% $$

### Maximal methane yield (MMY), experimental methane yield (EMY) and biodegradability (B_d_)

Maximal methane yield (MMY) of CS was calculated by Eqs (2) and (3)^20^, and B_d_ was analyzed according to Eq. (4)^21^.

2 $$\begin{array}{c}{{\rm{C}}}_{{\rm{n}}}{{\rm{H}}}_{{\rm{a}}}{{\rm{O}}}_{{\rm{b}}}{{\rm{N}}}_{{\rm{c}}}+(n-\frac{{\rm{a}}}{4}-\frac{{\rm{b}}}{2}+\frac{3{\rm{c}}}{4}){{\rm{H}}}_{2}{\rm{O}}\to (\frac{{\rm{n}}}{2}+\frac{{\rm{a}}}{8}-\frac{{\rm{b}}}{4}-\frac{3{\rm{c}}}{8}){{\rm{CH}}}_{4}+(\frac{{\rm{n}}}{2}-\frac{{\rm{a}}}{8}+\frac{{\rm{b}}}{4}+\frac{3{\rm{c}}}{8})\,{{\rm{CO}}}_{2}+{{\rm{cNH}}}_{3}\end{array}$$

3 $${\rm{MMY}}\,({\rm{mL}}\cdot {{\rm{gVS}}}^{-1})=\frac{22.4\times 1000\times (\frac{{\rm{n}}}{2}+\frac{a}{8}-\frac{b}{4}-\frac{3c}{8})}{12{\rm{n}}+{\rm{a}}+16{\rm{b}}+14{\rm{c}}}\times (1-{\rm{lignin}} \% -{\rm{ash}} \% )$$

4 $${{\rm{B}}}_{{\rm{d}}}=\frac{{\rm{EMY}}}{{\rm{MMY}}}\times 100 \% $$

### AD Experiments

The AD experiments were carried out in 500 ml digesters with an organic loading (OL) of 25.6 gVS·L^−1^ and feedstock/inoculum (F/I) ratio of 0.8. Distilled water was added to make the working volume up to 250 ml. Besides, two blank digesters, without feedstock which contained the same sludge and distilled water only, were used as corrections. Each digester was flushed for 3 min with 99.0% pure nitrogen to remove all the oxygen, screw cap and rubber stopper were fixed to ensure anaerobic condition. The bottles were then placed in an incubator at 37 °C for 61 days. All reactors were shaken manually for 1 min every day. The composition of biogas was analyzed once a day during the first ten days of digestion and three times a week later.

### Analytical methods

#### Basic characteristics

Total solid (TS) and volatile solid (VS) of CS and inoculum sludge were determined by the standard methods^22^. Elemental compositions (C, H, N, S) of CS and inoculum sludge were measured by an elemental analyzer (Vario EL cube, Germany). The oxygen content of CS was calculated by assuming C + H + O + N = 99.5% on a VS basis^23^. Cellulose, hemicellulose, and lignin content was determined by an A2000 fiber analyzer (ANKOM, USA) according to the Van Soest method^24^.

#### Methane yield

The daily biogas production was determined by measuring headspace pressure in each digester using 3151 WAL-BMP-Test system pressure gauge (WAL Mess-und Regelsysteme GmbH, Germany). The composition of biogas was determined by using a 7890 B gas chromatograph (Agilent, USA). Biogas production was calculated by Eq. (5)^25^.

5 $${{\rm{V}}}_{{\rm{biogas}}}=\frac{\,{\rm{\Delta }}{\rm{P}}\times {{\rm{V}}}_{{\rm{head}}}\times {\rm{C}}}{{\rm{R}}\times {\rm{T}}}$$

where V_biogas_ is daily biogas volume (L), P refers to absolute pressure (kPa), V_head_ represents volume of the head space (L), C is molar volume (22.4 L·mol^−1^), R means universal gas constant (8.314 J·mol^−1^·k^−1^), and T is equal to absolute temperature (K).

#### Pretreatment solution and biogas slurry

For pretreatment solution, the soluble chemical oxygen demand (COD) was determined by using HACH meter (DR2800 spectrophotometer). The soluble saccharides were measured by using phenol-sulfuric acid method with a spectrophotometer (UV-1800PC, Mapada, China) at 490 nm.

Total alkalinity (TA), total ammonia nitrogen (TAN), total volatile fatty acid (TVFA), pH value of the biogas slurry after digestion were analyzed. The pH value was measured with a le438 pH electrode (Mettler Toledo, USA). TAN was determined using HACH meter (DR2800 spectrophotometer). TA was observed by titrimetric method with 1.6 mol·L^−1^ of sulfuric acid. TVFA was measured by gas chromatography (GC) system (7890A, Agilent, USA).

### Kinetics model

In this study, the CMY has been modeled by employing modified Gompertz equation (6), which used to analyze the results of AD^26^.

6 $${\rm{B}}={{\rm{B}}}_{0}\exp \{-\exp [\frac{{{\rm{\mu }}}_{{\rm{m}}}{\rm{e}}}{{{\rm{B}}}_{0}}({\rm{\lambda }}-t)+1]\}$$

where B refers to the simulated cumulative methane production (mL·gVS^−1^), B_0_ represents the simulated highest methane yield (mL·gVS^−1^), μ_m_ stands for the maximum methane production rate (mL·gVS^−1^·day^−1^), e is equal to 2.72, λ is the lag phase time (day), and t means the digestion time (day).

### Micro-structure observation

#### XRD analysis

The crystallinity index (CrI) of the cellulose component, which was obtained under different pretreatment, was measured by X-ray diffraction (XRD). The Bruker D8-Advance (Germany) at 40 KV and 40 mA with Cu Kα radiation was used to examine the XRD patterns. Samples were scanned from 5 to 60° at a rate of 5°/min. CrI of these pretreated CS was calculated by Eq. (7)^27^.

7 $${\rm{CrI}}=[({{\rm{I}}}_{002}-{{\rm{I}}}_{{\rm{amorphous}}})/{{\rm{I}}}_{002}]\times 100 \% $$

where I_002_ is the maximal diffraction intensity of the 002 lattice plane, I_amorphous_ refers to the intensity of the background peak at 2θ = 18°.

#### FTIR analysis

The structural features of pretreated samples were measured by a Nicolet 6700 Fourier transform infrared (FTIR) spectrophotometer (Thermo Fisher Scientific, Waltham, MA, USA) with a DLATGS detector over a range of 400–4000 cm^−1^. Raw and pretreated CS were analyzed by grinding with KBr (1:100, w/w) and pressing into transparent pellets.

### Statistical analysis

All the data and graphs were processed using Origin Pro 8.0 (Origin Lab, USA). Analysis of variance (ANOVA) was used to statistically analyze the data.

## Results and Discussion

### Characterization of CS and inoculum

Table 1 shows the basis characteristics of raw CS and inoculum sludge. The VS/TS ratio of CS reached 96.75%, indicating a high organic matter, which was preferred for AD. Moreover, the content of cellulose and lignin was up to 50.42% and 16.32%, higher than some other widely used crop straw and biomass^21^. According to the data of the elements analysis in Table 1, the MMY of CS was calculated using Eqs (2) and (3) to be 362.9 mL·gVS^−1^.

**Table 1:** Basic characteristics of CS and inoculum^c^.

Parameter	Cotton stalk	Inoculum
TS (%)^a^	91.08 ± 0.00	9.66 ± 0.01
VS (%)^a^	88.12 ± 0.01	5.48 ± 0.03
Ash (%)^a^	2.96 ± 0.05	4.18 ± 0.04
VS/TS (%)	96.75 ± 0.01	56.71 ± 0.35
Cellulose (%)^b^	50.42 ± 0.86	ND
Hemicellulose (%)^b^	15.64 ± 0.03	ND
Lignin (%)^b^	16.32 ± 0.40	ND
C (%)^b^	45.96 ± 0.26	28.83 ± 0.66
H (%)^b^	5.97 ± 0.03	4.11 ± 0.05
O (%)^b^	43.50 ± 0.25	ND
N (%)^b^	1.02 ± 0.01	2.05 ± 0.08
S (%)^b^	0.09 ± 0.00	0.64 ± 0.05
C/N	45.06 ± 0.19	14.13 ± 0.84
pH	ND	7.58 ± 0.08
Calorific value (J·g^−1^)^b^	18447	ND

^c^Results are the average and their standard error of triplicate measurements. ^a^As total weight of sample; ^b^as TS of sample; ND = not detectable.

### Effects of different pretreatment methods on cumulative methane yield

**Figure 1:**
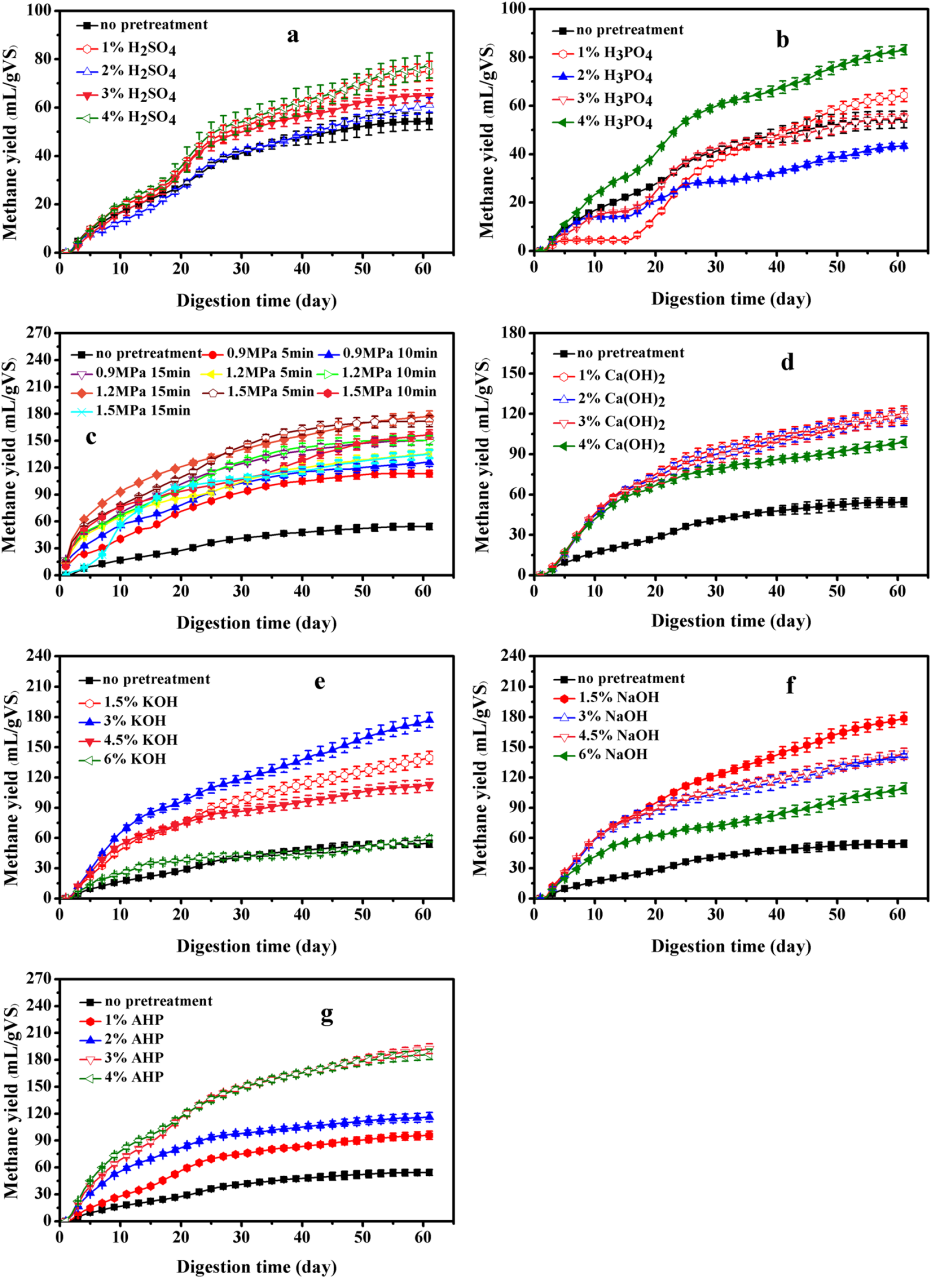
Methane yield of CS, (**a**) H_2_SO_4_-treated CS; (**b**) H_3_PO_4_-treated CS; (**c**) SE-treated CS; (**d**) Ca(OH)_2_-treated CS; (**e**) KOH-treated CS; (**f**) NaOH-treated CS; (**g**) AHP-treated CS.

Figure 1a and b show the CMY of H_2_SO_4_-treated CS and H_3_PO_4_-treated CS. In these groups, two different peaks of daily methane yield occurred during the digestion process of H_2_SO_4_-treated CS and H_3_PO_4_-treated CS, similar results were reported in the pretreatment of corn stover in anaerobic digestion process^9,28,29^. At the beginning, the daily methane yield of H_2_SO_4_-treated CS and H_3_PO_4_-treated CS quickly reached the first peak on 3^rd^ or 4^th^ day, which present in Figure S1 (supplementary data). The second peak emerged in H_2_SO_4_ treated CS on 22^nd^ day, whereas, in H_3_PO_4_ treated CS, it emerged on 19^th^, 20^th^ and 22^nd^ day (see Supplementary Fig. S1). The concentration of H_2_SO_4_ had almost no effect on the lag period, but the period was prolonged with the decrease of H_3_PO_4_ concentration. The results had no significant difference (P > 0.05) between 1% H_2_SO_4_-treated CS and 4% H_2_SO_4_-treated CS. Therefore, the suitable concentration of H_2_SO_4_ was regarded as 1%. From Fig. 1b, with the increase of the H_3_PO_4_ concentration, the CMY reached 83.1 mL·gVS^−1^ after 4% H_3_PO_4_ pretreatment, which had very significant difference (P < 0.05) compared to the CS pretreated by 1%, 2%, 3% H_3_PO_4_, respectively. Thus, the condition of 4% H_3_PO_4_ was the best choice.

The effect of pressure and time in SE pretreatment can be seen in Fig. 1c. The two highest cumulative methane production at 1.2 MPa for 15 min and 1.5 MPa for 5 min were 177.3 mL·gVS^−1^ and 171.8 mL·gVS^−1^ respectively, which showed no significant difference (P > 0.05), and improved 226.6%, 216.4% than the untreated CS. The maximum daily methane yield was 25.6 mL·gVS^−1^ on 2^nd^ day at 1.2 MPa for 15 min which was 7.5 times higher than the untreated CS (see Supplementary Fig. S1). Nevertheless, considering the costs and energy consumption, the pressure of 1.5 MPa for 5 min was the best choice in SE pretreatment of CS. When the pretreated pressure was 0.9 MPa and 1.2 MPa, the CMY increased with the rising of pretreatment time which was completely contrary to the data of 1.5 MPa. The difference indicated that some inhibitors might produce at the severe conditions. Moreover, SE pretreatment at 1.5 MPa for 15 min showed the peak of maximum daily methane yield on 9^th^ day which was quite different from other groups (see Supplementary Fig. S1). It also implied that the pretreatment process had some inhibitory effects on AD.

The CMY were 120.3 mL·gVS^−1^, 117.7 mL·gVS^−1^, 118.2 mL·gVS^−1^ from 1%, 2%, and 3% Ca(OH)_2_-treated of CS (Fig. 1d), which had no significant difference (P > 0.05) in CS pretreated by the concentration of 1%, 2%, 3%. The methane yield of KOH-treated CS at the concentration of 3% reached maximum at 177.1 mL·gVS^−1^, and improved 226.14% than the untreated CS (Fig. 1e). When the KOH concentration increased to 6%, the CMY reduced to 59.3 mL·gVS^−1^, which had no significant difference (P > 0.05) as compared to the untreated CS. This might be because many of the degradable organic matters in CS were dissolved by the pretreatment of 6% KOH. The maximal methane yield was found from 1.5% NaOH-treated CS (178.6 mL·gVS^−1^), which showed a highly significant (P < 0.01) reduction with increase of concentration of NaOH (Fig. 1f). It can be seen that the highest methane yield of CS pretreated by KOH and NaOH was superior to Ca(OH)_2_-treated CS. Considering the efficiency and cost of the pretreatment process in AD, 3%, 1.5%, 1% were an optimal concentration for KOH, NaOH, Ca(OH)_2_ pretreatment, respectively.

The CMY of CS pretreated by 3% AHP was up to 192.4 mL·gVS^−1^, which showed a remarkable difference (P < 0.01) compared with 96.0 mL·gVS^−1^ and 116.2 mL·gVS^−1^ from 1% and 2% AHP-treated CS (Fig. 1g). While the results of 3% AHP-treated CS and 4% AHP-treated CS were similar (P > 0.05), the optimum condition could be regarded as 3% for AHP pretreatment.

### Comparison of different pretreatments effect

#### The optimal results and kinetics of methane production after different pretreatments

**Figure 2:**
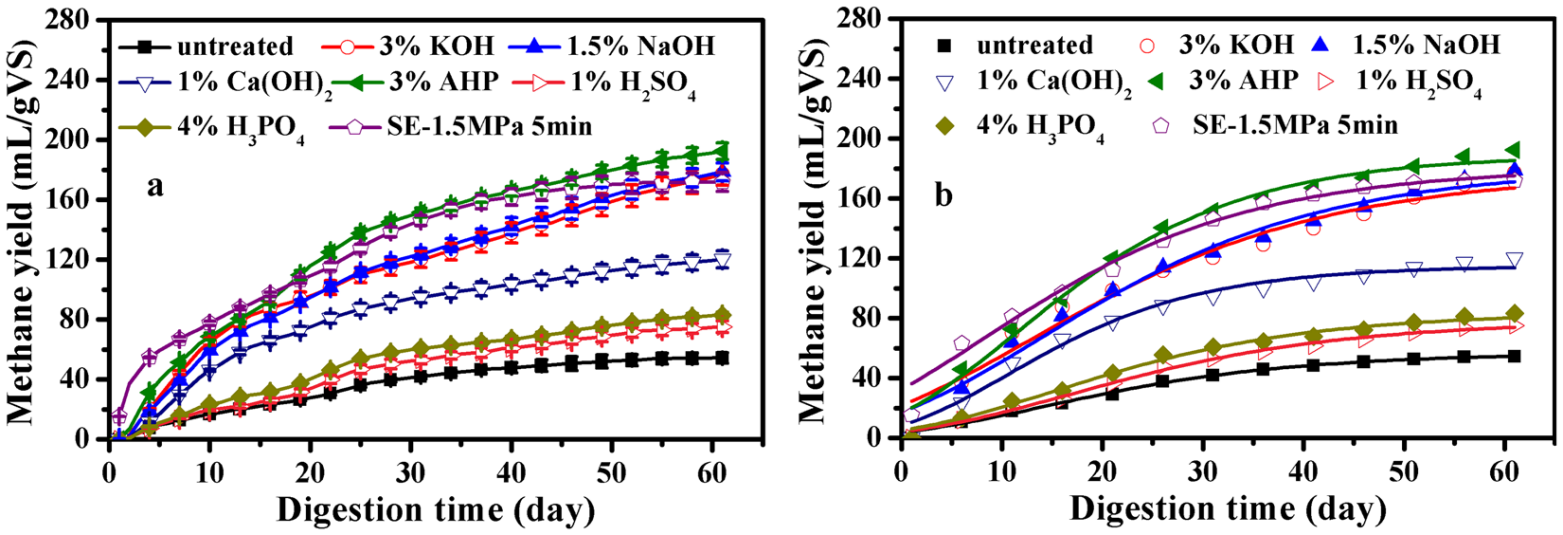
The optimal results and kinetics of methane yield after seven pretreatments.

The optimum results for each of the seven pretreatment processes are shown in Fig. 2a. The results showed that the highest CMY obtained from 3% AHP-pretreated CS, and improved 254.3% than the untreated CS. The results had no significant difference (P > 0.05) compared to SE pretreated CS at 1.5 MPa for 5 min, 3% KOH-treated CS, and 1.5% NaOH-treated CS. The CMY of 1% Ca(OH)_2_-treated CS was 47.3% and 48.5% lower than 3% KOH-treated CS and 1.5% NaOH-treated CS because Ca(OH)_2_ was less soluble in water. No significant difference (P > 0.05) within the CMY was observed between 1% H_2_SO_4_-treated CS and 4% H_3_PO_4_-treated CS. Obviously, the CMY of H_2_SO_4_ and H_3_PO_4_ treated CS was inferior to KOH, NaOH, Ca(OH)_2_, AHP, and SE pretreated CS. Moreover, the CMY of 2% H_3_PO_4_-treated CS reduced 20.1% than the untreated CS due to the long lag period. Therefore, the results implied that H_2_SO_4_ and H_3_PO_4_ pretreatment was not suitable for CS in AD process.

The selected optimum results were fitted by the Modified Gompertz model for statistical analysis (Fig. 2b). The kinetic parameters of the model are shown in Table 2. The correlation coefficients R^2^ values fell within the range of 0.967–0.995, respectively. It indicated that the Modified Gompertz model was well fitted to the CMY in all selected experimental groups. Moreover, the B_0_ values of the model were very close to the EMY at different pretreated conditions. The parameters of B_0_ and μ_m_ showed great enhancements than the untreated CS which illustrated that all pretreatment in this research could enhance the ultimate methane production and the maximum methane production rate. The start-up time of AD process is shown by the lag phase time (λ). Obviously, a lower λ means a short time of start-up. The λ of 1% H_2_SO_4_-treated CS and 4% H_3_PO_4_-treated CS was longer than the untreated CS, implying that a long lag period appeared which was consistent with the results of Fig. 1a,b. Compared with the untreated CS, the λ of other pretreated CS significantly reduced, especially CS pretreated at 1.5 MPa for 5 min which reduced from −0.21 to −6.97. The highest B_d_ values after pretreating by 3% AHP was 53.02% which implied that the digestion efficiency still had room to be further improved.

**Table 2:** The biodegradability of CS after pretreatment and kinetic parameters of the Modified Gompertz model.

Parameter	Modified Gompertz model				MMY (mL·gVS^−1^)	EMY (mL·gVS^−1^)	B_d_ (%)	Improved (%)
	B_0_	μ_m_	λ	R^2^				
untreated	56.6	1.46	−0.21	0.995	362.9	54.3	14.97	—
3% KOH	177.5	3.79	−4.45	0.967	362.9	177.1	48.81	226.14
1.5% NaOH	180.6	4.10	−2.41	0.982	362.9	178.6	49.23	228.95
1% Ca(OH)_2_	114.9	3.84	−0.43	0.983	362.9	120.3	33.14	121.44
1% H_2_SO_4_	78.2	1.85	1.04	0.991	362.9	75.2	20.72	38.49
4% H_3_PO_4_	83.7	2.11	0.37	0.990	362.9	83.1	22.91	53.09
3% AHP	189.1	5.46	−1.45	0.991	362.9	192.4	53.02	254.28
SE-1.5 MPa 5 min	180.7	4.38	−6.97	0.991	362.9	171.8	47.35	216.39

#### Characteristics of pretreated CS, pretreatment solution and biogas slurry

Table 3 shows the composition of treated CS, the component of pretreatment solution and the characteristics of the biogas slurry after AD. The content of cellulose of all treated CS improved apart from SE pretreated CS at 1.5 MPa for 5 min, whereas AHP pretreated CS has shown the greatest improvement, rising about 7.32%. Furthermore, because a greater amount of non-lignocellulosic substances dissolved, the content of total fiber also increased except SE treated CS. Compared with the untreated CS, the fraction of hemicellulose slightly enhanced in 1% H_2_SO_4_-treated CS and 4% H_3_PO_4_-treated CS. On contrary, in alkaline treated CS it reduced. The content of cellulose and hemicellulose decreased sharply from 50.42% to 46.80% and 15.64% to 4.66% after SE pretreatment at 1.5 MPa for 5 min which indicated that SE pretreatment could destroy the structure of CS severely and remove more lignocellulose than other pretreatment.

**Table 3:** Characteristics of pretreated CS, pretreatment solution after pretreatment and biogas slurry after AD^a^.

Pretreatment	COD (mg·L^−1^)	Soluble saccharides (mg·L^−1^)	pH	TA (mgCaCO_3_·L^−1^)	TAN (mg·L^−1^)	TVFA (mg·L^−1^)	Cellulose (%)	Hemicellulose (%)	Total fiber (%)
untreated	—	—	7.66	6200	1310	0	50.42 ± 0.86	15.64 ± 0.03	82.38 ± 1.31
3% KOH	13880	5110	7.36	4560	1095	0	56.65 ± 0.34	14.50 ± 0.02	90.64 ± 0.48
1.5% NaOH	14220	4440	7.37	4300	1210	0	56.70 ± 0.06	14.80 ± 0.12	90.84 ± 0.10
1% Ca(OH)_2_	6120	920	7.50	5720	1265	0	53.29 ± 0.18	15.09 ± 0.21	85.97 ± 0.68
1% H_2_SO_4_	4300	980	7.65	5940	1340	0	54.25 ± 0.51	16.01 ± 0.43	87.66 ± 0.41
4% H_3_PO_4_	1340	150	7.66	5860	1215	0	52.81 ± 0.41	16.04 ± 0.09	86.20 ± 0.23
3% AHP	5760	580	7.64	6160	1310	0	57.74 ± 0.72	15.52 ± 0.23	88.99 ± 0.44
SE-1.5 MPa 5 min	—	—	7.67	7020	1775	0	46.80 ± 0.38	4.66 ± 0.75	69.98 ± 0.60

^a^Results are the average and their standard error of triplicate measurements.

The COD and soluble saccharides were the indicators of degradable and solubilized lignin and carbohydrate in pretreatment solution^30^. From Table 3, the concentration of COD and saccharides followed the same trends. However, the value of COD and soluble saccharides showed significant difference, which indicated that they contained some other organic compounds such as aromatic acids, ethanol, furfural derivatives, and uronic acids in pretreatment solution^18^. The two highest COD concentrations appeared in 3% KOH and 1.5% NaOH loading in the black liquor were 13880 mg·L^−1^ and 14220 mg·L^−1^, respectively, which indicated that KOH and NaOH could remove a large quantity of lignin and carbohydrate. The COD and soluble saccharides of 3% AHP pretreatment were 5760 mg·L^−1^ and 580 mg·L^−1^ in the liquid, less than KOH, NaOH, Ca(OH)_2_ pretreatment, implying that AHP pretreatment could decrease the loss of degradable organic matters. Obviously, the pretreatment solution of KOH, NaOH, Ca(OH)_2_ and AHP treated CS had much more COD and soluble saccharides than H_2_SO_4_ and H_3_PO_4_ treated CS. The results indicated that more lignin and carbohydrate could be deconstructed by alkali pretreatment compared with acid pretreatment.

The digestion process stability was shown by the characteristics of pH values, TA, TAN, and TVFA in biogas slurry after AD (Table 3). The pH values of all pretreatment were in range of 7.36–7.67, which were suitable for the growth of bacteria. TA indicates the buffering capacity to neutralize acids, which could protect against the low pH of AD system. Some researches held that the TAN was one of the most important indicators in the process of AD because the ammonia could cause the accumulation of VFA, which resulted in a sharp drop of pH^31^, and then affected the activity of microbes. However, the pH value did not drop and accumulation of TVFA and inhibition of microbial activity were not observed in this study. Besides, the concentration of TAN in blank which contained the same amount of sludge and water was about 1500 mg·L^−1^. Therefore, effluent parameters collectively suggested that the AD system after different pretreatments were stable, and pretreatment didn’t influence the anaerobic digestion system stablity.

#### Analysis of modifications in the chemical structure followed by different pretreatments

**Figure 3:**
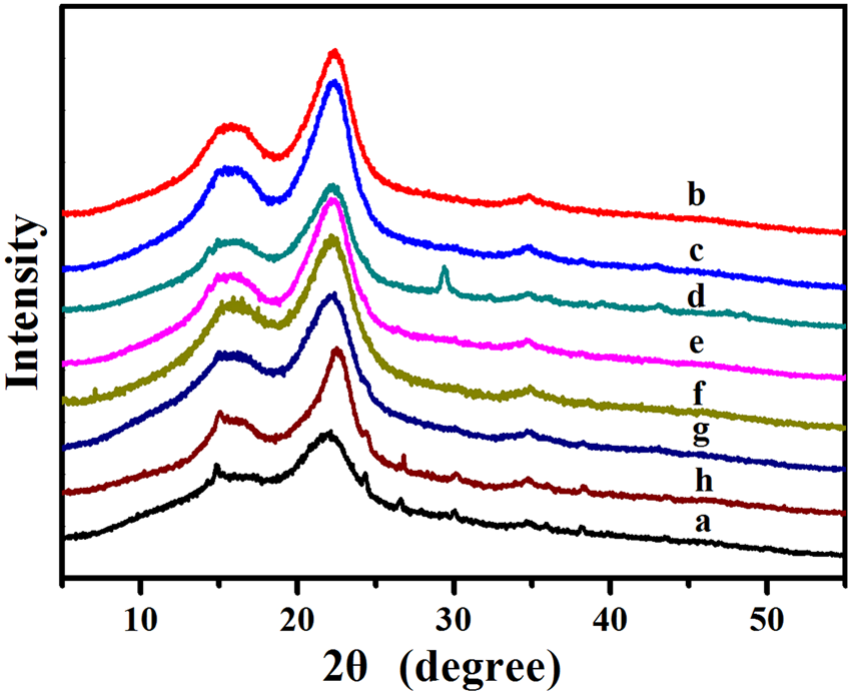
XRD patterns, (**a**) untreated CS; (**b**) 3% KOH-treated CS; (**c**) 1.5% NaOH-treated CS; (**d**) 1% Ca(OH)_2_-treated CS; (**e**) 3% AHP-treated CS; (**f**) 1% H_2_SO_4_-treated CS; (**g**) 4% H_3_PO_4_-treated CS; (**h**) SE-treated CS at 1.5 MPa for 5 min.
- FTIR Analysis
  The FTIR spectra of raw CS and pretreated CS was shown in Fig. 4. The wide band at around 3418.5 cm^−1^ is linked with the level of intermolecular and intramolecular hydrogen bonding^33^, whereas, the peak at 2920.0 cm^−1^ with shoulder bands is due to aliphatic CH_2_ and symmetric and asymmetric CH_3_, respectively^14,33^. The absorbance at 1739.2 cm^−1^ was owing to the ester groups, indicating the sign existed in lignin^12^. After KOH, NaOH, Ca(OH)_2_ and AHP pretreatment, this peak vanished, implying that alkaline condition could destroy ester groups, and then cut down the linkage in the lignin-carbohydrate. The strong and wide band at 1244.4 cm^−1^ was ascribed to the =C-O in the aromatic ether group confirming lignin prescence^18^. The absorbance disappeared in KOH-treated CS, NaOH-treated CS, Ca(OH)_2_-treated CS and AHP-treated CS and decreased in H_2_SO_4_-treated CS, H_3_PO_4_-treated CS, SE-treated CS, implying that all pretreatments are excellent in removing lignin, especially alkaline pretreatment. Furthermore, the intensity at 900 cm^−1^ band corresponding to β-D-cellulose linkages had a significant decrease in all pretreatments, which indicated the removal of amorphous components.

- XRD Analysis
  XRD was employed to determine and calculate the crystallinity index of cellulose after pretreatment (Fig. 3). There existed similar peaks of raw CS and pretreated CS at 18°, 22° and 35°, which were obtained from cellulose I^32^, illustrated that cellulose crystalline allomorph did not change after the pretreatment. The CrI of untreated CS was 34.1% and it increased to 50.4%, 51.3%, 51.2%, and 47.0% after 3% KOH, 1.5% NaOH, 3% AHP, and SE pretreatment at 1.5 MPa for 5 min, respectively. This was probably because a great proportion of non-crystalline components, including lignin and hemicellulose, were removed as a result of these pretreatments. An increase in the portion of exposed crystalline structure was noticed in the pretreated samples, hence, the CrI of pretreated CS improved compared with the untreated CS. These results were in conformity with the previous studies^7,12,16^.

**Figure 4:**
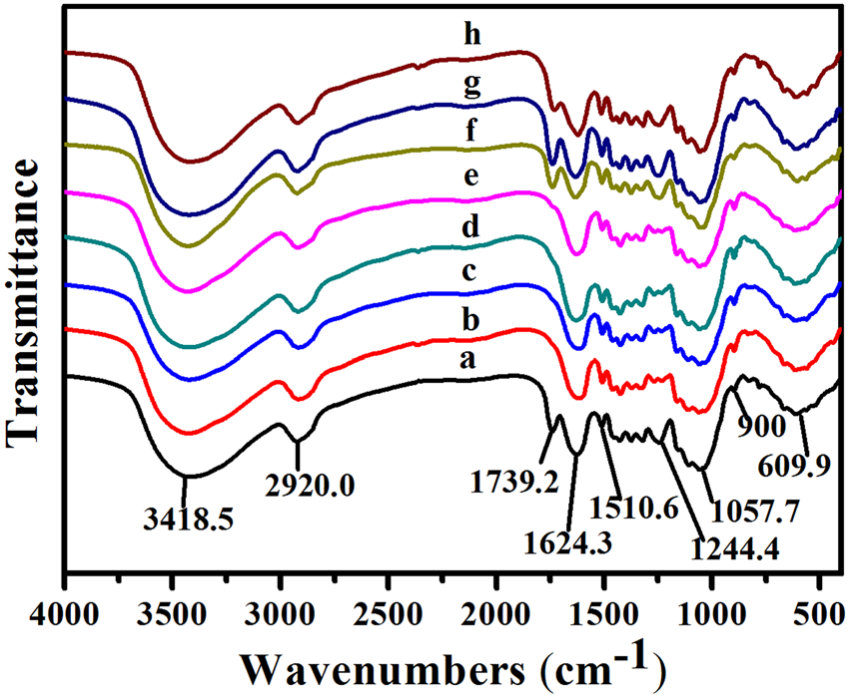
FTIR spectra, (**a**) untreated CS; (**b**) 3% KOH-treated CS; (**c**) 1.5% NaOH-treated CS; (**d**) 1% Ca(OH)_2_-treated CS; (**e**) 3% AHP-treated CS; (**f**) 1% H_2_SO_4_-treated CS; (**g**) 4% H_3_PO_4_-treated CS; (**h**) SE-treated CS at 1.5 MPa for 5 min.

### Practical application

The performances of pretreatment by H_2_SO_4_ and H_3_PO_4_ were inferior to the other pretreatments. Moreover, the methane yields of CS could be influenced by the long lag period. Therefore, the results showed that H_2_SO_4_ or H_3_PO_4_ might not be suitable for pretreating CS during anaerobic digestion process. SE pretreatment has lower pollution, however it is not highly recommended, due to higher energy consumption. Besides, many inhibitory compounds such as furfural and HMF might be generated from SE pretreatment process^34^. The appropriate conditions such as pressure and time should be chosen, otherwise the process might influence the subsequent digestion reactions. The compared results showed that KOH and NaOH were both suitable for pretreating CS in AD process. But Na^+^ ion from the pretreatment of NaOH might influence the digestion process, causing the inhibition of methanogenesis^10^. Moreover, the liquid after NaOH pretreatment could result in serious environment problems such as soil salinization and water pollution^10^. On contrary, the waste liquid containing KOH might have environmental benefits, including the production of fertilizer^10,30^. KOH is much more expensive than Ca(OH)_2_ and NaOH. Therefore, it is possible to consider that using combined KOH and Ca(OH)_2_ alkaline pretreatment to reduce the cost by decreasing the KOH dosage and maintaining the efficiency of anaerobic digestion^16^. Compared with NaOH pretreatment, AHP pretreatment could considerably decrease the dosage of NaOH, and reduce Na^+^ ion pollution. Furthermore, hydrogen peroxide in the pretreatment solution could be easily decomposed into water and oxygen to avoid secondary pollution. Therefore, AHP pretreatment was considered to be the promising method for treating CS.

## Conclusion

This study compared the performance of various pretreated CS in anaerobic digestion. Acid treated CS could not achieve high methane yield due to the long lag period. Therefore, H_2_SO_4_ and H_3_PO_4_ are not suitable to pretreat CS in AD process. Although SE pretreatment has given high methane yield, but the treatment is not suitable due to the higher energy consumption and lack of availability of the industrial scale equipment, because it is hard to build big continuous machine with low cost. The efficiency of AD process after KOH and NaOH pretreatment was better than Ca(OH)_2_ pretreatment. Results indicated that CS could efficiently be converted to methane after AHP pretreatment at concentration of 3%. In summary, AD after AHP pretreatment could be an effective method to improve the CS utilization. Besides, this study offers a systemic insight into the biogas productions from pretreated CS and provides useful information on the pretreatment of CS in a cost effective and environment friendly way.

## Electronic supplementary material

Supplementary Information

## References

[1] Yuan X (2016). Enhancing anaerobic digestion of cotton stalk by pretreatment with a microbial consortium (MC1). Bioresour Technol..

[2] Du SK (2013). High pressure assist-alkali pretreatment of cotton stalk and physiochemical characterization of biomass. Bioresour Technol..

[3] Song Z (2014). Comparison of seven chemical pretreatments of corn straw for improving methane yield by anaerobic digestion. PLoS One..

[4] Keshav PK, Shaik N, Koti S, Linga VR (2016). Bioconversion of alkali delignified cotton stalk using two-stage dilute acid hydrolysis and fermentation of detoxified hydrolysate into ethanol. Ind Crop Prod..

[5] He Y (2009). Investigation on the Changes of Main Compositions and Extractives of Rice Straw Pretreated with Sodium Hydroxide for Biogas Production. Energ Fuel..

[6] Zhang Q, Tang L, Zhang J, Mao Z, Jiang L (2011). Optimization of thermal-dilute sulfuric acid pretreatment for enhancement of methane production from cassava residues. Bioresour Technol..

[7] Wang M (2016). Bioethanol production from cotton stalk: A comparative study of various pretreatments. Fuel..

[8] Sambusiti C, Ficara E, Rollini M, Manzoni M, Malpei F (2012). Sodium hydroxide pretreatment of ensiled sorghum forage and wheat straw to increase methane production. Water Sci Technol..

[9] Tian Y (2016). Research on anaerobic digestion of corn stover enhanced by dilute acid pretreatment: Mechanism study and potential utilization in practical application. J Renew Sustain Ener..

[10] Zheng Y, Zhao J, Xu F, Li Y (2014). Pretreatment of lignocellulosic biomass for enhanced biogas production. Prog Energ Combust..

[11] Cheng X-Y, Zhong C (2014). Effects of Feed to Inoculum Ratio, Co-digestion, and Pretreatment on Biogas Production from Anaerobic Digestion of Cotton Stalk. Energ Fuel..

[12] Li J (2015). Enhancing methane production of corn stover through a novel way: sequent pretreatment of potassium hydroxide and steam explosion. Bioresour Technol..

[13] Mirahmadi K, Kabir MM, Jeihanipour A, Karimi K, Taherzadeh MJ (2010). Alkaline pretreatment of spruce and birch to improve bioethanol and biogas production. Bioresources..

[14] Nieves DC, Karimi K, Horváth IS (2011). Improvement of biogas production from oil palm empty fruit bunches (OPEFB). Ind Crop and Prod..

[15] Lopez Torres M, Mdel EL (2008). C. Effect of alkaline pretreatment on anaerobic digestion of solid wastes. Waste Manag..

[16] Li L (2015). Pretreatment of Corn Stover for Methane Production with the Combination of Potassium Hydroxide and Calcium Hydroxide. Energ Fuel..

[17] Bondesson PM, Dupuy A, Galbe M, Zacchi G (2015). Optimizing ethanol and methane production from steam-pretreated, phosphoric acid-impregnated corn stover. Appl Biochem Biotechnol..

[18] Sun C (2015). Impacts of Alkaline Hydrogen Peroxide Pretreatment on Chemical Composition and Biochemical Methane Potential of Agricultural Crop Stalks. Energ Fuel..

[19] Zheng M, Li X, Li L, Yang X, He Y (2009). Enhancing anaerobic biogasification of corn stover through wet state NaOH pretreatment. Bioresour Technol..

[20] Feng J (2017). Enhanced methane production of vinegar residue by response surface methodology (RSM). AMB Express..

[21] Chen X, Gu Y, Zhou X, Zhang Y (2014). Asparagus stem as a new lignocellulosic biomass feedstock for anaerobic digestion: increasing hydrolysis rate, methane production and biodegradability by alkaline pretreatment. Bioresour Technol..

[22] APHA (1992). Standard Methods for the Examination of Water and Wastewater.

[23] Rincón B, Heaven S, Banks CJ, Zhang Y (2012). Anaerobic Digestion of Whole-Crop Winter Wheat Silage for Renewable Energy Production. Energ Fuel..

[24] Soest PJV, Robertson JB, Lewis BA (1991). Methods for Dietary Fiber, Neutral Detergent Fiber, and Nonstarch Polysaccharides in Relation to Animal Nutrition. J Dairy Sci..

[25] Liu X, Zicari SM, Liu G, Li Y, Zhang R (2015). Pretreatment of wheat straw with potassium hydroxide for increasing enzymatic and microbial degradability. Bioresour Technol..

[26] Kafle GK, Kim SH (2013). Anaerobic treatment of apple waste with swine manure for biogas production: Batch and continuous operation. Appl Energ..

[27] Li Y (2014). Thermophilic Solid-State Anaerobic Digestion of Alkaline-Pretreated Corn Stover. Energ Fuel..

[28] Chen G (2010). Experimental co-digestion of corn stalk and vermicompost to improve biogas production. Waste Manag..

[29] Ashekuzzaman SM, Poulsen TG (2011). Optimizing feed composition for improved methane yield during anaerobic digestion of cow manure based waste mixtures. Bioresour Technol..

[30] Liu X, Zicari SM, Liu G, Li Y, Zhang R (2015). Improving the bioenergy production from wheat straw with alkaline pretreatment. Biosyst Eng..

[31] Chen Y, Cheng JJ, Creamer KS (2008). Inhibition of anaerobic digestion process: a review. Bioresour Technol..

[32] Sun Y (2007). Hydrolysis of cotton fiber cellulose in formic acid. Energ Fuel..

[33] Rodríguez-Abalde Á (2013). Study of thermal pre-treatment on anaerobic digestion of slaughterhouse waste by TGA-MS and FTIR spectroscopy. Waste Manage Res..

[34] Barakat A, Monlau F, Steyer JP, Carrere H (2012). Effect of lignin-derived and furan compounds found in lignocellulosic hydrolysates on biomethane production. Bioresour Technol..

