# Supplementary material for: Enhancement of methane production from Cotton Stalk using different pretreatment techniques
Source: Sci Rep. 2018 Feb 22;8:3463. doi: 10.1038/s41598-018-21413-x (PMC5823884; doi:10.1038/s41598-018-21413-x)
Supplement: Supplementary file 1 — Supplementary Information [file 41598_2018_21413_MOESM1_ESM.doc]

**Enhancement of methane production from Cotton Stalk using different pretreatment techniques**

Han Zhang^1^, Zhifang Ning^2^, Habiba Khalid^2^, Ruihong Zhang^2,3^, Guangqing Liu^2^, Chang Chen^2, *^

^1^ Biomass Energy and Environmental Research Center, College of Life Science and Technology, Beijing University of Chemical Technology, Beijing, 100029, China

^2^ Biomass Energy and Environmental Research Center, College of Chemical Engineering, Beijing University of Chemical Technology, Beijing, 100029, China

^3^ Department of Biological and Agricultural Engineering, University of California, Davis, CA 95616, United States

^*^ [chenchang@mail.buct.edu.cn](mailto:chenchang@mail.buct.edu.cn)


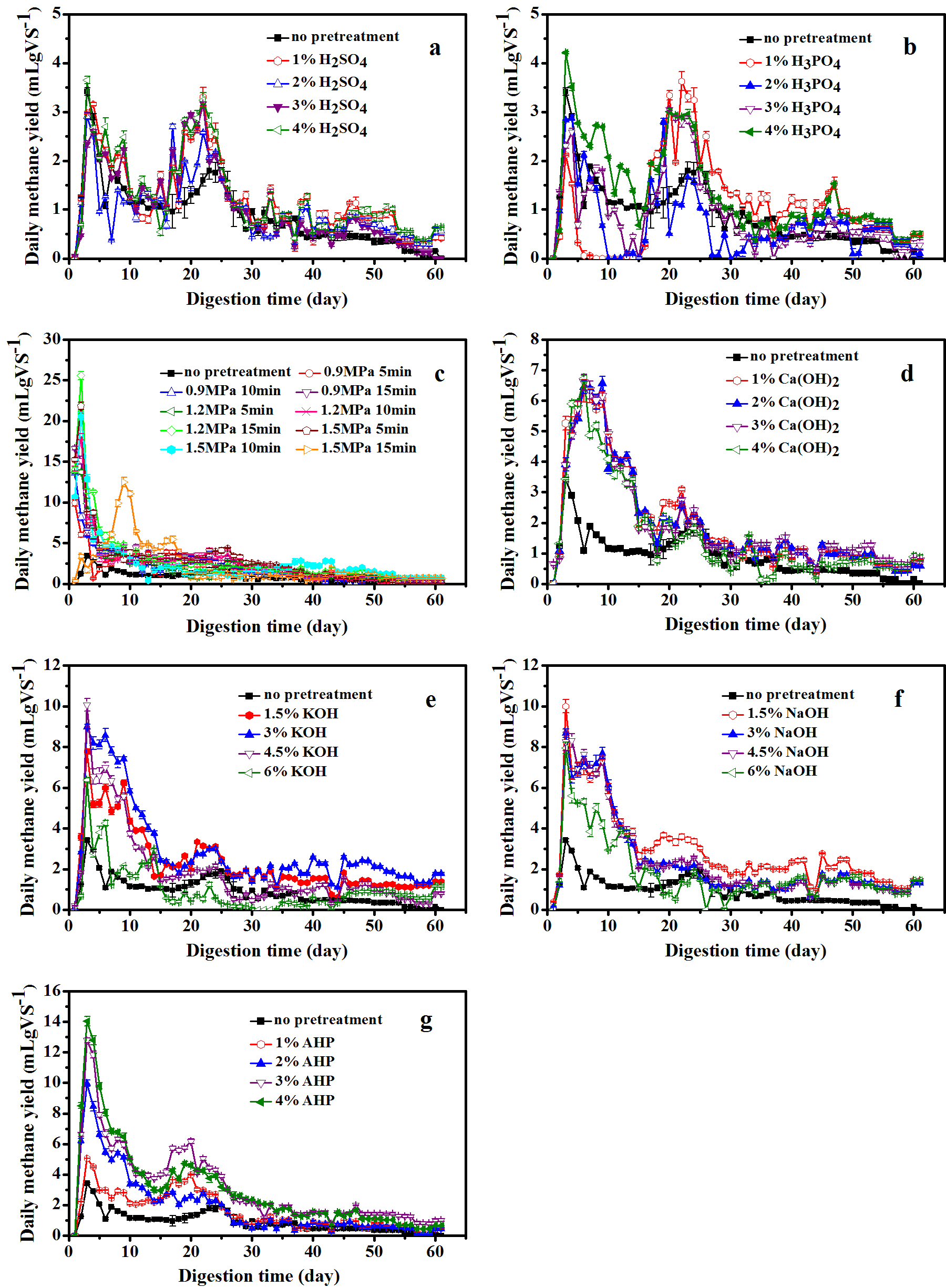


Figure S1. Daily methane yield of CS, (a) H_2_SO_4_-treated CS; (b) H_3_PO_4_-treated CS; (c) SE-treated CS; (d) Ca(OH)_2_-treated CS; (e) KOH-treated CS; (f) NaOH-treated CS; (g) AHP-treated CS.
